# Purification and Biological Function of Caldecrin

**DOI:** 10.3390/medicines8080041

**Published:** 2021-07-23

**Authors:** Akito Tomomura, Kenjiro Bandow, Mineko Tomomura

**Affiliations:** 1 Division of Biochemistry, Department of Oral Biology & Tissue Engineering, Meikai University School of Dentistry, 1-1 Keyakidai, Sakado, Saitama 350-0283, Japan; kbando@dent.meikai.ac.jp; 2 Department of Oral Health Sciences, Meikai University School of Health Sciences, 1-1 Akemi, Urayasu, Chiba 279-8550, Japan; mineko-t@dent.meikai.ac.jp

**Keywords:** calcium metabolism, bone metabolism, protease, osteoclast, macrophage, RANKL, LPS, TLR4, TREM2

## Abstract

Blood calcium homeostasis is critical for biological function. Caldecrin, or chymotrypsin-like elastase, was originally identified in the pancreas as a serum calcium-decreasing factor. The serum calcium-decreasing activity of caldecrin requires the trypsin-mediated activation of the protein. Protease activity-deficient mature caldecrin can also reduce serum calcium concentration, indicating that structural processing is necessary for serum calcium-decreasing activity. Caldecrin suppresses the differentiation of bone-resorbing osteoclasts from bone marrow macrophages (BMMs) by inhibiting receptor activator of NF-κB ligand (RANKL)-induced nuclear factor of activated T-cell cytoplasmic 1 expression via the Syk–PLCγ–Ca^2+^ oscillation-calcineurin signaling pathway. It also suppresses mature osteoclastic bone resorption by RANKL-stimulated TRAF6–c-Src–Syk–calcium entry and actin ring formation. Caldecrin inhibits lipopolysaccharide (LPS)-induced osteoclast formation in RANKL-primed BMMs by inducing the NF-κB negative regulator A20. In addition, caldecrin suppresses LPS-mediated M1 macrophage polarization through the immunoreceptor triggering receptor expressed on myeloid cells (TREM) 2, suggesting that caldecrin may function as an anti-osteoclastogenic and anti-inflammatory factor via TREM2. The ectopic intramuscular expression of caldecrin cDNA prevents bone resorption in ovariectomized mice, and the administration of caldecrin protein also prevents skeletal muscle destruction in dystrophic mice. In vivo and in vitro studies have indicated that caldecrin is a unique multifunctional protease and a possible therapeutic target for skeletal and inflammatory diseases.

## 1. Introduction

Calcium homeostasis is controlled by calcium absorption in the intestine and reabsorption along the renal tubules, as well as by bone formation and resorption. Calcium homeostasis is regulated by parathyroid hormone (PTH) and thyroid gland-derived calcitonin, and activated vitamin D_3_ produced in the kidney. In addition, the pancreas is involved in calcium metabolism. Hypocalcemia is frequently observed in acute pancreatitis [1]. Glucagon [2,3], amylin [4,5], and calcitonin gene-related peptides [6,7] secreted from the pancreas may be responsible for hypocalcemia. While the exact pathological mechanisms of hypocalcemia are unknown, the pancreas may secrete hypocalcemic factors. We previously purified and cloned the hypocalcemic factor, caldecrin, from the pancreas [8,9,10]. In this review article, we discuss the roles of the multifunctional protease, caldecrin, in the pancreas.

## 2. Purification and Cloning of Caldecrin

**Figure 1:**
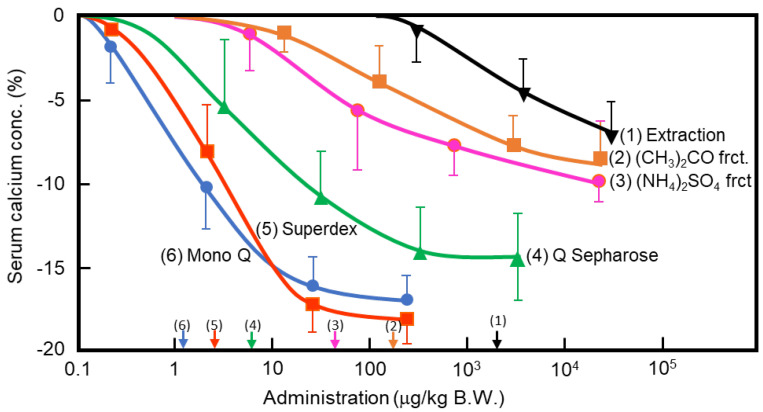
Purification of porcine caldecrin and its serum calcium-decreasing activity. The caldecrin was isolated from porcine pancreas following the following purification steps: porcine acetone powder was extracted (1), and active fraction was separated with acetone (CH_3_)_2_CO precipitation (2), saturated ammonium sulfate (NH_4_)_2_SO_4_ precipitation (3), and then Q Sepharose Fast–Flow ion-exchange chromatography (4), Superdex 75 size–exclusion fast protein liquid chromatography (FPLC) (5), and Mono Q ion–exchange FPLC (6). Dose–dependent curves of serum calcium decreased activity and its half maximal effective concentration (EC50) values, as shown by arrows on the *x*-axis, were prepared from the representative preparation.

In the 1960s, Takaoka et al. first demonstrated that a pancreatic extract of porcine had hypocalcemic activity [11,12]. In 1992, Tomomura et al. purified a hypocalcemic factor, calcium-decreasing factor (caldecrin), from a porcine pancreatic extract [8]. Caldecrin was purified from acetone powder of porcine pancreas via fractionation with acetone (30–60%) and saturated ammonium sulfate (45–60%), followed by ion-exchange chromatography on Q Sepharose Fast–Flow (pH 5.5), gel filtration on Superdex 75 fast protein liquid chromatography (FPLC), and ion-exchange chromatography on Mono Q FPLC (pH 5.5). The isolated caldecrin was confirmed as a single peak by high-performance liquid chromatography. To identify caldecrin, each fraction of the purification steps was injected into the tail vein of fasted mice, and serum calcium concentrations were measured 4 h post-injection. The serum calcium concentration decreased in a dose-dependent manner as the products of subsequent purification steps were administered, and the hypocalcemic activity increased as a result of the purification process (Figure 1). In addition to this in vivo experiment, the ability of caldecrin to inhibit PTH-stimulated calcium release was assessed using Ca^45^-prelabeled fetal mouse long bone organ cultures. Caldecrin inhibited PTH-stimulated Ca^45^ release from the bone to the culture medium at concentrations as low as 10 ng/mL. These experiments showed that caldecrin is an anionic protein (pI: 4.5) with a molecular weight of approximately 28 kDa. In addition, we showed that caldecrin is a serine protease with chymotryptic activity [8]. The immature form of caldecrin (procaldecrin), which is purified in the presence of the serine protease inhibitor diisopropyl fluorophosphate (DFP) from porcine pancreas, is activated by trypsin treatment in a dose- and time-dependent manner, giving rise to the activated caldecrin that exerts chymotryptic activity [9].

In 1995, we first isolated rat caldecrin cDNA by immunoscreening with an anti-caldecrin antibody [10]. The nucleotide sequence was almost identical to that of a PCR clone named rat elastase IV (ELA4) [13]. A frame shift caused by a minor nucleotide change in both genes resulted in the difference of the amino acid sequences of the central region of caldecrin from that of ELA4. Thus, the lysine-X bond of purified rat caldecrin was digested with a metal endopeptidase in the presence of the chymotrypsin inhibitor phenylmethylsulfonyl fluoride (PMSF). The partial amino acid sequence of caldecrin fragments purified from rat pancreas completely matched that encoded by cDNA. Furthermore, the partial amino acid sequence of purified porcine caldecrin was closely related to that of the corresponding fragments of purified rat caldecrin. However, the amino acid sequence of rat and porcine caldecrin differed from the deduced amino acid sequence of rat ELA4 cDNA. Amino acid sequences of caldecrin showed higher homology with elastase than chymotrypsinogen A and B, although the N-terminal amino acid sequence of caldecrin revealed that the mature form has a disulfide-linked activation peptide, which is characteristic of chymotrypsin. In 1995, Gomis-Rüth et al. reported the crystalline structure of bovine chymotrypsinogen C [14]. The amino acid sequence of chymotrypsinogen C was very close to that of rat caldecrin, suggesting a high degree of similarity between caldecrin and chymotrypsin C (CTRC).

In 1996, human caldecrin genes were cloned [15]. We transfected the insect cell line Sf9 with a recombinant baculovirus harboring human caldecrin cDNA. Recombinant human caldecrin was purified from the culture medium by using hydroxyapatite column chromatography. Subsequently, the purified recombinant human caldecrin showed hypocalcemic activity. To address whether ELA4 is transcribed and translated in vivo and has proteolytic activity, we constructed rat ELA4 cDNA by combinational PCR and compared the recombinant rat ELA4 with the recombinant rat caldecrin synthesized in a baculovirus expression system [16]. We detected recombinant caldecrin protein in the medium. However, in the case of ELA4, we could not detect ELA4 protein in the cells and the medium. Furthermore, we detected caldecrin mRNA expression in rat pancreas but no ELA4, suggesting that ELA4 might be a single nucleotide polymorphism of the caldecrin gene. It is now known that chymotrypsin C, caldecrin, and ELA4 are the same protein encoded by the CTRC gene. The CTRC gene is located on chromosome 1p36.21 of the human genome [17]. The rat and mouse CTRC genes are located on 5q36 and 4E1 of each genome, respectively [18].

## 3. Protein Structure and Protease Activity of Caldecrin

### 3.1. Structure of Chymotrypsin C (Caldecrin)

**Figure 2:**
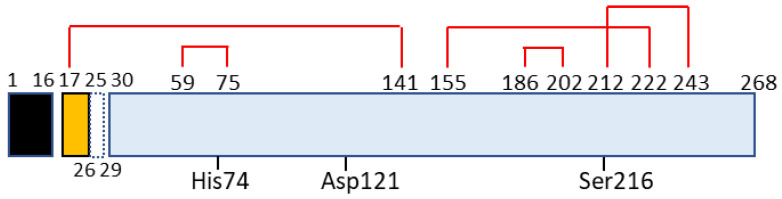
Schematic for domain structure of caldecrin. Black box (signal peptide: amino acid residue number 1–16); yellow box (pro-peptide:17–29 including 26–29 or 27–29 peptide removed by autoactivation); blue box (mature protein: 30–268); red lines (disulfide bridges with cysteine number); His74, Asp121, and Ser216 (charge relay system for serine protease activity).

Chymotrypsin C, also termed caldecrin, is a 268-amino acid-long protein. Its sequence comprises a signal peptide (16 amino acids long, from residues 1 to 16), pro-peptide (13 amino acids, residues 17–29), and mature protein (239 amino acids, residues 30–268). The crystal structure of bovine Ctrc [14] revealed that rat and human caldecrin have five disulfide bridges: Cys17–Cys141, Cys59–Cys75, Cys155–Cys222, Cys186–Cys202, and Cys212–Cys243 [10,15] (Figure 2).

Chymotrypsin C possesses two barrel structures, between which the charge-relayed catalytic triad (His74, Asp121, and Ser216) is located. The activation peptide is first cleaved at the Arg29–Ile30 peptide bond by trypsin, and further cleaved at Asp25–Leu26 [10] or Leu26–Ser27 [19] by the autoactivation of chymotrypsin C. The cleaved Cys17–Asp25 or Cys17–Leu26 long peptide remains attached to the mature protein by a disulfide bridge such as Cys17–Cys141, a structure that resembles chymotrypsin [10,14,15,19,20].

### 3.2. Proteolytic Activity and Specificity of Chymotrypsin C (Caldecrin)

The enzyme classification of chymotrypsin C (caldecrin) is *EC 3.4.21.2*. It shows hydrolytic activity that can cleave leucyl, tyrosyl, phenylalanyl, methionyl, tryptophanyl, glutamine, and asparagine bonds. Chymotrypsin C preferentially hydrolyzes leucyl bonds compared to chymotrypsin A [21]. Humans have five other chymotrypsin-like elastase genes that encode the structurally similar proteins chymotrypsin-like elastase family, member 1 (CELA1, *EC3.4.21.36*), pancreatic CELA2A and 2B (*EC3.4.21.71*), and pancreatic CELA3A and 3B (*EC3.4.21.70*). The protease activity of CELA2 preferentially cleaves leucine, methionyl, and phenylalanyl residues and hydrolyzes elastin [22]. CELA3B preferentially cleaves alanyl residues, but has little elastolytic activity [23]. Human caldecrin was more similar to CELA2A (63.4%), 2B (59.6%), 3A (52.2%), and 3B (53.0%) than with chymotrypsin B (42.5%) [15]. The altered protease activity of chymotrypsin C revealed that it can be a risk factor for chronic pancreatitis, a role that is described in detail in Section 3.3.

### 3.3. Chymotrypsin C (Caldecrin) and Pancreatitis

Chronic pancreatitis is a progressive inflammatory disease of the pancreas. It is characterized by acinar cell atrophy, fibrotic tissue replacement, and duct irregularities with calcifications [24]. The pathological mechanism of pancreatitis is uncontrolled trypsin activity [25]. Cationic trypsinogen (PRSS1) mutations are gain-of-function mutations that stimulate the autoactivation of the proform to trypsin, which are associated with autosomal dominant hereditary pancreatitis [26]. Loss-of-function mutations in serine protease inhibitor Kazal-type 1 (SPINK1), which can inactivate intrapancreatic trypsin activity, are associated with pancreatitis risk [27]. Therefore, the inactivation of irregularly produced intrapancreatic PRSS1 by SPINK1 or by an unidentified serine protease (Rinderknecht’s enzyme Y) has been proposed to protect against pancreatitis [28,29,30]. In 2006, Nemoda and Sahin-Tóth reported that chymotrypsin C (caldecrin) stimulates the autoactivation of human cationic trypsinogen [31]. In 2007, Szmola and Sahin-Tóth reported that the unidentified enzyme Y was identified as chymotrypsin C (caldecrin), in which the main role of chymotrypsin C is trypsinogen activation and trypsin degradation [32]. In 2008, Rosendahl et al. reported that loss-of-function variants in the CTRC gene were risk factors for chronic pancreatitis [33]. Masson et al. also identified a CTRC mutation in patients with idiopathic chronic pancreatitis [34]. Thus, loss-of-function mutations in CTRC can cause a decrease in the catalytic activity of CTRC and impaired trypsinogen degradation, which are causative risk factors for chronic pancreatitis [35]. CTRC is also a susceptibility gene for tropical calcific pancreatitis associated with calcium deposition in the pancreas [36,37]. For genetic risk factors in chronic pancreatitis, see the www.pancreasgenetics.org (accessed on 28 May 2021) website [38].

## 4. Non-Proteolytic Functions of Caldecrin

### 4.1. Caldecrin and Calcium Metabolism

**Figure 3:**
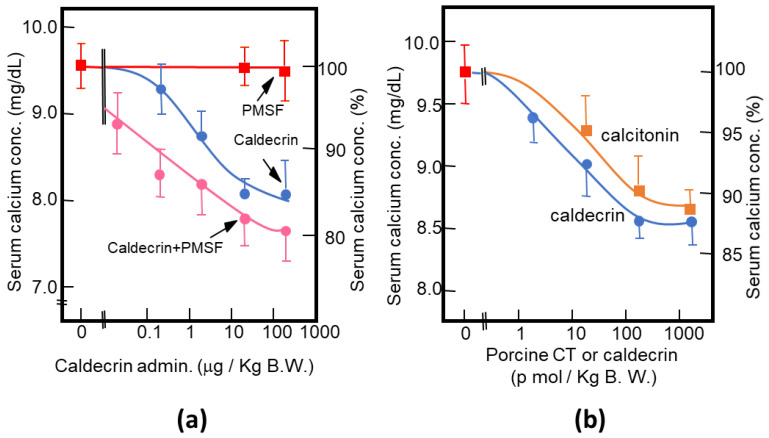
Characterization of the serum calcium-decreasing activity of caldecrin. (**a**) PMSF treatment does not inhibit the serum calcium-decreasing activity of activated caldecrin; (**b**) comparison of the serum calcium decreasing activities of porcine caldecrin and calcitonin.

As described above, purified porcine and rat caldecrin from the pancreas and produced recombinant rat and human caldecrin protein decreased serum calcium concentration in mice. Caldecrin dose-dependently decreased the serum calcium concentration. The effect resulted in a maximum decrease of 15–20% with 20–100 μg (about 0.7–3.5 nmol)/kg mice body weight. Procaldecrin did not exhibit serum calcium-decreasing activity, but acquired serum calcium-decreasing and protease activity after trypsin treatment. PMSF treatment after the trypsin activation of procaldecrin abolished its protease activity but did not affect the serum calcium-decreasing activity [8] (Figure 3a). The calcium-decreasing activity of porcine caldecrin was almost the same as that of porcine calcitonin (1 nmol/kg body weight) (Figure 3b). Pretreatment with PMSF or recombinant caldecrin with point mutations at positions coding for activity-related amino acids (Hm: His74Ala or Sm: Ser216Ala substitution) decreased serum calcium concentration in vivo and bone destruction activity in vitro and abolished the protease activity of caldecrin. Caldecrin not only decreased calcium concentration but also hydroxyproline serum concentration, which is a marker of bone resorption, suggesting that caldecrin inhibits bone destruction by osteoclasts [10]. Therefore, the mechanism by which caldecrin inhibits osteoclast formation and/or function remains to be investigated.

In 1996, Izbicka et al. independently purified a calcium metabolism-regulating factor from a porcine pancreas by determining the inhibitory effect of the proliferation of human osteosarcoma MG-63 cells and bone resorption in organ culture stimulated by PTH [39]. The factor had a molecular weight of 28 kDa, and it showed 92% homology with human elastase IIIB (CELA3B) in the N-terminus. Recombinant human elastase IIIB inhibited bone resorption in organ cultures stimulated with 1,25-dihydroxyvitamin D_3_. This anti-resorptive activity was abolished by PMSF treatment, highlighting the importance of the proteolytic activity of elastase IIIB in the inhibition of bone resorption. The differences between the hypocalcemic mechanisms of caldecrin and elastase IIIB have not yet been elucidated.

### 4.2. Caldecrin and Osteoclast

#### 4.2.1. Caldecrin and RANK Signaling

**Figure 4:**
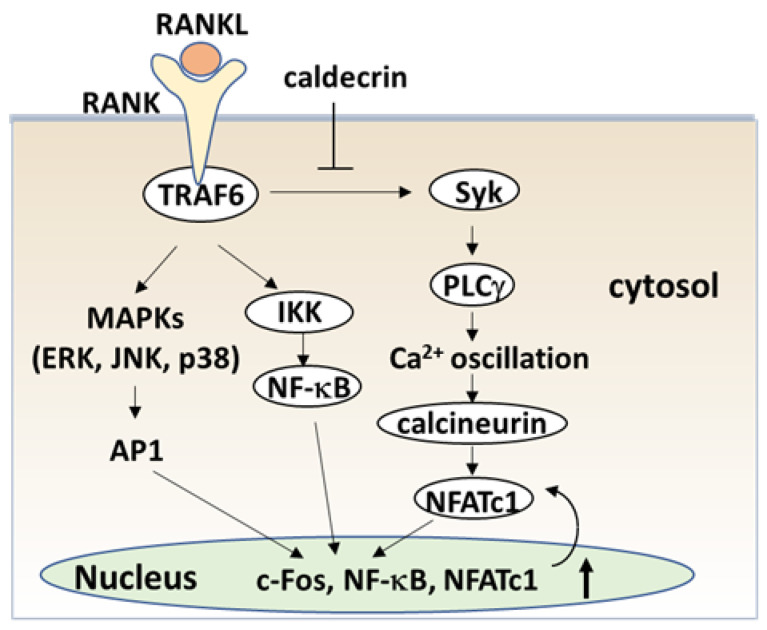
Caldecrin inhibits RANKL signaling in the initial stage of osteoclast differentiation. RANKL binding to RANK activates both NF-κB and MAPKs, such as ERK, JNK, and p38. Activation of NF-κB and MAPK signals is then transmitted to c-Fos and NFATc1 induction. Caldecrin inhibits RANK-stimulated phosphorylation of Syk and PLCγ, abolishing Ca^2+^ oscillation and activation of calcineurin, and amplification of NFATc1 in the initial stage of osteoclast differentiation. AP-1: activator protein-1; ERK: extracellular signal-regulated kinase; IKK: IκB kinase; JNK: C-Jun N-terminal kinase; MAPK: mitogen-activated protein kinase; NFATc1, nuclear factor of activated T-cells cytoplasmic 1; NF-κB: nuclear factor-κB; PLCγ: phospholipase Cγ; RANK: receptor activator of NF-κB; RANKL: RANK ligand; Syk: spleen tyrosine kinase; TRAF6: tumor necrosis factor receptor-associated factor 6.

The serum calcium concentration is affected by osteoclast activity. Osteoclasts execute bone resorption, which is differentiated from bone marrow by key molecules such as macrophage colony-stimulating factor (M-CSF) and receptor activator of nuclear factor-kappa B (NF-κB) ligand (RANKL) [40,41,42,43,44]. Osteoclast differentiation and maturation occur in the following stages: (i) osteoclast precursor cells are produced from bone marrow cells in response to M-CSF and begin to differentiate following stimulation by RANKL; and (ii) osteoclasts fuse with each other to form multinucleated giant cells. Multinucleated cells secrete protons and cathepsin K, which are required for bone resorption. Osteoclast differentiation is tightly regulated by many molecules to maintain bone homeostasis [45,46,47]. (Figure 4). During the initial stage, RANKL binds to its receptor RANK, which induces the recruitment of the adaptor protein, tumor necrosis factor receptor-associated factor 6 (TRAF6) [48]. Activated TRAF6 stimulates NF-κB by activating IκB kinase (IKK) [49]. TRAF adaptor proteins also activate mitogen-activated protein kinases (MAPKs) such as extracellular signal-regulated kinase (ERK), C-Jun N-terminal kinase (JNK), and p38 [50,51,52,53]. NF-κB and MAPK signaling activates activator protein-1 (AP-1) including c-Fos, which in turn activates the master transcription factor in osteoclastogenesis, nuclear factor of activated T-cell cytoplasmic 1 (NFATc1) [54,55,56].

RANK signaling also activates phospholipase Cγ (PLCγ)-dependent Ca^2+^ signaling through splenic tyrosine kinase (Syk) [57,58,59]. Finally, calcineurin, a calcium/calmodulin-dependent serine/threonine phosphatase activated by intracellular Ca^2+^ concentration, causes the dephosphorylation of NFATc1 and induces translocation from the cytoplasm into the nucleus [60]. Thus, TRAF6, NF-κB, c-Fos and NFATc1 are required for the initiation stage of RANKL-induced osteoclast differentiation (Figure 4).

To understand the serum calcium-decreasing effects of caldecrin on osteoclast-mediated bone resorption, we investigated whether caldecrin inhibits the initial stage of osteoclast differentiation following RANKL exposure. In murine bone marrow macrophages and macrophage-derived RAW264.7, protease-deficient caldecrin inhibited RANKL-stimulated osteoclast differentiation [61]. The macrophage-type colonies formed from BMCs in the absence or presence of caldecrin were not different, suggesting that caldecrin does not affect macrophage formation. The frequency of osteoclast progenitor formation in the presence of M-CSF alone was not different from that in the presence of M-CSF and caldecrin. Thus, caldecrin did not affect macrophage colony formation or osteoclast progenitors from BMCs. However, caldecrin suppressed RANKL-stimulated mononuclear osteoclast differentiation, assessed by tartrate-resistant acid phosphatase (TRAP) staining and enzymatic activity, a specific osteoclast enzyme commonly used as a marker. Caldecrin inhibited the RANKL-induced phosphorylation of Syk and PLCγ and abolished Ca^2+^ oscillations within 5–10 min of caldecrin exposure. Caldecrin inhibited the activation of calcineurin, a protein that enhances NFATc1 activity. Finally, caldecrin inhibited the RANKL-stimulated nuclear translocation of NFATc1 and its mRNA accumulation, whereas other RANKL-stimulated transcription factors such as NF-κB κ and c-Fos were unaffected. Thus, we found that caldecrin inhibits osteoclast differentiation by suppressing NFATc1 activity via the RANKL-mediated calcium signaling pathway at the initial stage of osteoclastogenesis (Figure 4).

**Figure 5:**
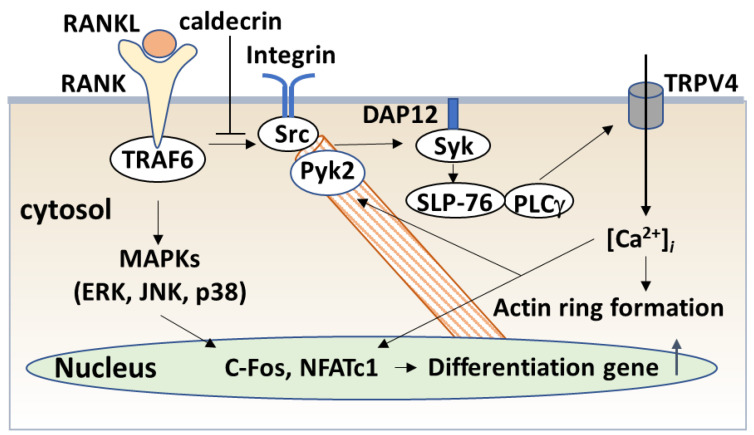
Caldecrin inhibits RANKL signaling in mature osteoclasts. RANKL–RANK binding activates c-Fos and NFATc1. RANK also activates c-Src and the c-Src–Syk complex. Activated Syk phosphorylates PLCγ via SLP-76, which leads to the activation of TRPV4 channels and evokes Ca^2+^ influx. Increased Ca^2+^ levels activate Pyk2 and are associated with Src, leading to cytoskeletal organization. Caldecrin inhibits RANKL-induced phosphorylation of c-Src, Syk, PLCγ, SLP-76, and Pyk2 in mature osteoclasts. Caldecrin also abolishes Ca^2+^ entry into the cytoplasm through the TRPV4 channel and TRAF6–c-Src interaction. Akt: AKR mouse thymoma kinase; Src, sarcoma; SLP-76:SH2 domain containing leukocyte protein of 76kDa; TRPV4: Transient Receptor Potential Vanilloid 4.

In the late stage of osteoclastogenesis, amplified NFATc1 induces the expression of osteoclast-specific genes, leading to osteoclast differentiation (Figure 5). Mature osteoclasts create a unique cytoskeletal structure, termed the sealing zone, which consists of an actin ring attached to the bone surface [62,63]. The integrin vitronectin receptor αvβ3 binds to vitronectin present in the bone matrix, inducing the recruitment of c-Src tyrosine kinase to the integrin receptor. Activated c-Src phosphorylates Syk, which phosphorylates the DNAX-activating protein of 12 kDa (DAP12) [64], and SLP-76, which induces cytoskeletal organization and bone resorption [65]. Calcium-dependent proline-rich tyrosine kinase (PYK2) is an adhesion kinase localized in the sealing zone, which is activated by binding to αvβ3 integrin and subsequent phosphorylation by Src kinase [66]. TRAF6-induced cytoskeletal changes are mediated by interactions with cytoplasmic c-Src [67]. Thus, RANKL–RANK signaling enhances the TRAF6–c-Src interaction, which activates the formation of the Src–Syk and Src–Pyk2 complexes that induce the cytoskeletal organization of mature osteoclasts.

Calcium signaling pathways have been shown to play a role in bone resorption, exerting effects on actin metabolism, cytoskeletal organization, and cell–matrix interactions. RANKL signaling activates PLCγ and enhances the production of inositol trisphosphate (IP3), which results in the release of Ca^2+^ from the ER through transient receptor potential vanilloid channel 2 (TRPV2), which subsequently causes oscillations in Ca^2+^ concentration [68]. Ca^2+^ oscillations disappear during differentiations and are replaced by RANKL-evoked Ca^2+^ influx via TRPV4 and 5 [69,70]. Thus, RANKL-triggered Ca^2+^ influx in multinucleated osteoclasts through TRPV channels maintains NFATc1 activity and activates Pyk2, which is essential for actin filament organization (Figure 5).

Next, we investigated whether caldecrin inhibits RANKL-induced mature osteoclast function. Caldecrin inhibited RANKL-stimulated osteoclastic bone resorption in vitro, but did not induce apoptosis [71]. In addition, caldecrin inhibited the RANKL-induced phosphorylation of c-Src, Syk, PLCγ, SLP-76, and Pyk2 in mature osteoclasts but not the phosphorylation of ERK, JNK, and Akt. Furthermore, caldecrin inhibited RANKL-induced Ca^2+^ entry through TRPV4 and actin ring formation in mature osteoclasts, RANKL-stimulated c-Src kinase activity, and integrin–c-Src–Syk association and RANKL-mediated TRAF6–c-Src association. Thus, we found that caldecrin suppresses RANKL-mediated Ca^2+^ signaling and actin ring formation in mature osteoclasts via suppression of the TRAF6–c-Src-–Syk signaling pathway, resulting in the suppression of bone resorption (Figure 5).

In this section, we conclude that protease activity-deficient caldecrin inhibits both RANKL-stimulated osteoclast formation from bone marrow progenitors and pre-existing mature osteoclastic bone resorption, resulting in the serum calcium-decreasing activity of caldecrin in vivo. Next, we investigated the role of caldecrin in inflammation-induced bone loss.

#### 4.2.2. Caldecrin and TLR4 Signaling 

Inflammation is known to cause bone loss. Bacterial lipopolysaccharide (LPS), a major constituent of the outer membrane of Gram-negative bacteria, is a potent inducer of bone loss in inflammatory diseases, including periodontal disease, bacterial arthritis, and dental implant infections [72,73]. Toll-like receptor (TLR) family members, which are proteins homologous to the Drosophila Toll protein, play a critical role in the innate immune system. TLR (TLR1–9) is expressed in osteoclast progenitors, of which TLR2 and 4 are also expressed in osteoclasts [74]. LPS has been shown to stimulate osteoclast formation and bone resorption in vivo through TLR4 [75,76].

The signaling cascade of TLR4 has been extensively studied in macrophages [77,78,79]. LPS induces inflammation upon the production of pro-inflammatory cytokines, such as interleukin-1 (IL-1) β, TNF-α, and IL-6 in macrophages and lymphocytes. Activated TLRs, except for TLR3, induce pro-inflammatory cytokine production through the canonical myeloid differentiation factor 88 (MyD88), which recruits TRAF6 downstream and activates IKK and the NF-κB pathway [80], leading to osteoclast formation in vitro. Although LPS is known to induce bone loss in vivo, LPS can both inhibit and stimulate osteoclastogenesis in vitro. The simultaneous activation of TLR4 and RANK signaling by LPS and RANKL, respectively, inhibits osteoclast formation in BMMs [74,81]. In this context, LPS/TLR4 activates NF-κB, p38, ERK1/2, and JNK, but inhibits RANKL-induced Nfatc1 expression. In contrast, LPS treatment enhanced the osteoclast differentiation of BMMs primed with M-CSF and RANKL [81,82]. The expression of Nfatc1 in RANKL-committed preosteoclasts is no longer affected by subsequent LPS treatment [82]. Therefore, RANKL-primed NFATc1 expression is a prerequisite for LPS-stimulated osteoclast formation.

RANKL/RANK and LPS/TLR4 signaling pathways in osteoclast formation share TRAF6, a ubiquitin E3 ligase, and downstream signaling pathways such as NF-κB activation. The LPS response is regulated by negative feedback with an NF-κB-inducible A20, which is a deubiquitinating protease encoding tumor necrosis factor alpha-induced protein 3 (*TNFAIP3*) [83]. A20 removes lysine 63 (K63)-linked polyubiquitin chains from TRAF6 and promotes K48-polyubiquitination for proteasomal degradation; thus, NF-κB-stimulated A20 plays an anti-inflammatory role by inhibiting IκB phosphorylation and NF-κB activation [84]. LPS induces osteoclast formation from RANKL-pretreated macrophages and the expression of A20, which is associated with TRAF6 degradation and NF-κB inhibition [85]. The overexpression of A20 inhibits osteoclastogenesis in a TRAF6-dependent manner, whereas the silencing of A20 restores TRAF6 expression and NF-κB activation, resulting in LPS-enhanced bone resorption [86]. Thus, the induction of NFATc1 by RANKL–TRAF6 is necessary before the increase in the levels of A20 by LPS. Therefore, the anti-inflammatory molecule A20 acts as a barrier to uncontrolled activation during osteoclast differentiation.

We investigated whether caldecrin inhibited LPS-induced osteoclastogenesis. Osteoclast progenitors from mouse BMMs and RAW264.7 cells were primed with a low dose of RANKL for 40 h and subsequently exposed to LPS in the absence of RANKL, which caused osteoclast formation [87]. LPS stimulated the phosphorylation of ERK, JNK, p38, and IκB. Furthermore, LPS stimulated the expression of osteoclast differentiation markers, such as ACP5 (tartrate-resistant acid phosphatase 5), CTSK (cathepsin K), and DCSTAMP (dendrocyte expressed seven transmembrane protein) in RANKL-primed RAW264.7 cells and osteoclast progenitors. When RANKL priming was combined with protease-deficient caldecrin treatment, caldecrin inhibited the LPS-stimulated phosphorylation of IκB and that of JNKs, MAPKs, ERKs, and p38 to a lesser extent, leading to the inhibition of the marker gene expression.

Interestingly, pretreatment with RANKL and caldecrin increased A20 mRNA and protein levels. Furthermore, a reduction in the levels of A20 by means of RNA interference (RNAi) in RAW264.7 cells pretreated with caldecrin and RANKL resulted in enhanced osteoclast formation in response to LPS stimulation. These results indicate that caldecrin enhances A20 expression at the RANKL priming stage, which interferes with LPS-evoked NF-κB activation. Caldecrin alone did not activate the IκB, ERKs, JNKs, and p38 signaling pathways, unlike LPS, suggesting that A20-induced caldecrin may be an anti-inflammatory protein. The mechanism of A20 induction by caldecrin was further elucidated.

#### 4.2.3. Caldecrin and TREM2 Signaling

Recent studies revealed that tissue macrophages, including osteoclasts, express DAP12 and its pairing triggering receptor on myeloid cell 2 (TREM-2), and participates in diverse cell processes, including osteoclastogenesis, inflammation [88,89,90]. TREM-2 in mouse macrophages and RAW264.7 cells stimulated by anti-TREM-2 antibody cross-linking enhanced RANKL-stimulated osteoclast formation, whereas silencing TREM-2 resulted in the inhibition of bone differentiation, indicating that TREM-2 is a positive regulator of osteoclast differentiation and function [91]. DAP12-deficient mice also show impaired osteoclastogenesis in vitro [92]. In contrast, TREM2-deficient mice show accelerated osteoclastogenesis in vitro [93,94]. There are conflicting results regarding the relationship between TREM-2, DAP12, and osteoclastogenesis in humans and mice, suggesting that TREM-2′s contribution to osteoclasts’ biology may vary depending on the influence of other receptors such as TREM-1 and/or on the presence of TREM-2 ligands with variable avidity/affinity; for example, complete or partial DAP12 phosphorylation by TREM-2 ligand binding may induce either activating or inhibitory signaling through TREM-2/DAP12 [93].

TREM2/DAP12 signaling contributes to macrophage activation. Tissue macrophages have two key functions: (1) to interact with pathogens such as LPS and modulate the adaptive immune responses, and (2) to facilitate tissue repair and tissue regeneration. These macrophage polarizations, categorized as M1 and M2, are modulated by the chemokine system [95,96,97]. Macrophages activated by LPS or interferon-γ alone or in combination are differentiated as classical M1 activation, which produces pro-inflammatory cytokines, whereas Th2-related cytokines IL-4 or IL-13, and anti-inflammatory molecules such as IL-10 and TGF-β, promote alternative M2 activation, which shows an anti-inflammatory and pro-healing phenotype [97,98]. TREM-2/DAP12 signaling contributes to the negative regulation of LPS/TLR4-mediated M1 macrophage polarization [99]. TREM-2-deficient macrophages enhanced the expression of pro-inflammatory cytokines and suppressed phagocytosis following TLR4 stimulation with LPS, demonstrating that TREM-2 suppresses inflammation and promotes bacterial clearance.

**Figure 6:**
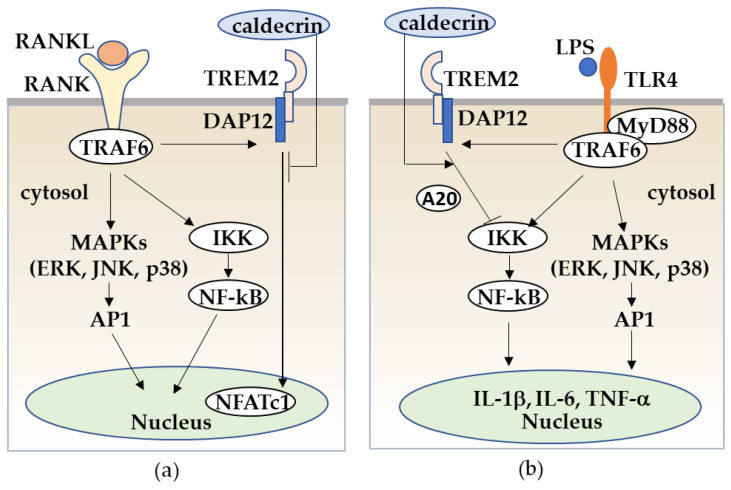
Caldecrin inhibits RANKL/RANK stimulated osteoclastogenesis from macrophages (**a**) and LPS/TLR4-stimulated macrophage activation through TREM-2 (**b**). (**a**) RANKL/RANK-stimulated osteoclast differentiation from macrophages is promoted with the activation of MAPKs, NF-κB, and the TREM-2/DAP12 axis. Caldecrin inhibits the RANKL/RANK/TRAF6 co-stimulatory TREM-2/DAP12 signal which is critical for NFATc1 activation. (**b**) LPS/TLR4 stimulates the TRAF6/NF-kB and MAPK pathways in macrophages, resulting in the activation of macrophages to M1 polarization with increased production of pro-inflammatory cytokines such as TNF-α, IL-1, and IL-6. Caldecrin inhibits LPS-induced M1 macrophage polarization through TREM-2/DAP12 signaling with the induction of A20.

To address whether caldecrin inhibits osteoclast formation via TREM-2, we prepared TREM-2 gene knockout (KO) RAW264.7 cells. Based on the study showing that TREM-2/DAP12 signaling is essential for RANKL-induced osteoclastogenesis, TREM-2-KO RAW264.7 cells were impaired to differentiate them into osteoclasts following RANKL stimulation. Therefore, to elucidate the effect of caldecrin on LPS-induced M1 macrophage polarization through TREM-2, BMMs and TREM-2-KO RAW 264.7 cells were incubated with LPS and IFN-γ [100]. LPS induced the phosphorylation of p38, JNKs, and ERKs, the degradation of IκB and the expression of pro-inflammatory cytokines such as IL-1β, IL-6, and TNF-α in mouse BMMs, whereas caldecrin suppressed LPS-induced IκB degradation and pro-inflammatory cytokine production but did not affect p38, JNKs, and ERKs signaling pathways. Caldecrin also inhibited M1 macrophage polarization in BMMs stimulated with LPS and IFN-γ. In RAW264.7 cells, caldecrin also inhibited LPS-induced IκB degradation, pro-inflammatory cytokine expression, and M1 macrophage polarization, while in Trem2-KO RAW264.7, caldecrin-mediated suppression was not observed. These results suggest that caldecrin is a negative regulator of LPS-induced inflammatory responses via TREM2. Taken together, our findings suggest that the inhibitory mechanism of caldecrin in RANKL/RANK-mediated osteoclast formation and LPS/TLR4-mediated inflammation in macrophages relies, at least in part, on TREM2. However, this is a mere hypothesis and requires further testing (Figure 6).

### 4.3. Caldecrin and Inflammation-Related Diseases

Rheumatoid arthritis (RA) is an autoimmune disease characterized by osteoclast-mediated bone and cartilage destruction resulting from inflammation in the synovium [101,102,103]. Osteoclast precursor cells are identified in areas of pannus invasion into the bone in RA. RANKL is expressed by both synovial fibroblasts and activated T lymphocytes derived from synovial tissues from patients with RA [104,105,106]. Pro-inflammatory cytokine levels, including TNF-α, IL1α, IL-1β, and IL-6, induce RANKL expression in synovial fibroblasts in RA, resulting in the enhancement of osteoclastogenesis in RA [107,108,109,110]. The A20 is decreased in monocytes and synovium from RA patients, suggesting that A20 may have a protective role in RA [111,112,113]. 

We investigated whether caldecrin could improve inflammation-related bone diseases. Therefore, we investigated whether caldecrin suppresses RANKL expression in synovium derived from patients with RA. TNF-α treatment increased RANKL expression in synovial fibroblasts from patients with RA but not in those from healthy individuals [114]. Caldecrin inhibited TNF-α-stimulated RANKL overexpression in RA fibroblasts, suggesting that caldecrin inhibits inflammatory cytokine-induced RANKL expression in RA.

Osteoporosis is associated with estrogen deficiency and bone loss in postmenopausal women. The decrease in bone mass is due to enhanced or imbalanced bone resorption by osteoclasts vs. osteoblastic bone formation in osteoporosis [115,116]. While basal levels of RANKL and M-CSF are essential for physiological osteoclast formation, T-cell-derived pro-inflammatory cytokines, such as TNF-α, are responsible for the upregulation of osteoclast formation in estrogen deficiency [117,118]. The ovariectomy fails to induce bone loss in TNF-α-deficient mice and in p55 TNF receptor KO mice [119]. Ovariectomized mice are an animal model commonly used to study postmenopausal osteoporosis, as they exhibit increased serum calcium levels due to elevated bone resorption.

To address whether caldecrin improves OVX-induced osteoporosis, we transfected plasmids encoding wild-type caldecrin or the protease-deficient mutant caldecrin in the femoral muscle of OVX model mice [120]. Caldecrin abolished changes in calcium serum concentration and collagen degradation in OVX mice, and restored bone resorption parameters to normal levels by micro-CT analysis, which decreased the bone surface to bone volume ratio, trabecular separation, increased bone volume density, and trabecular thickness and number, indicating that caldecrin suppresses estrogen deficiency-induced osteoporosis.

These findings, taken together with our in vitro experiments, suggest caldecrin as a possible therapeutic target in arthritis and osteoporosis.

### 4.4. Caldecrin and Muscular Dystrophy

Takaoka et al. [11,12] administered pancreatic extract to patients diagnosed with myasthenia gravis and muscular dystrophy, including patients with fascio-scapulo-humeral muscular dystrophy (FSHD). FSHD is an autosomal dominant disease, and it is the third most common muscular dystrophy (1:15,000 to 1:20,000). It is characterized by weakness of the skeletal muscles of the face, shoulders, and upper arms. The symptoms often progress towards the lower body, and in the latest stages of the disease, the humeral, truncal, and leg muscles are also affected [121]. Takaoka et al. reported that extract administration improved the symptoms of FSHD, suggesting that the hypocalcemic effect of the pancreatic extract could contribute to slowing down the progression of muscular dystrophy [12]. In 2005, Lefkowitz D.L. and Lefkowitz S.S. reported that Ca^2+^-triggered TNF-α induction, and the overexpression of adenine nucleotide translocator-1 protein, which is a component of the mitochondrial permeability transition pore, was observed in FSHD [122]. They used the Ca^2+^ channel blocker, diltiazem, for the treatment of FSHD, resulting in the prevention of the progression of muscle wasting, and proposed the use of diltiazem and a TNF-α inhibitor for the treatment of FSHD. In 2011, we investigated the effect of caldecrin in a naturally occurring mutant model of human congenital muscular dystrophy, a dy/dy mouse model. These mice lacked the laminin gene and exhibited defective muscle basement membranes. The peritoneal administration of caldecrin protein or the muscular ectopic expression of caldecrin improved the muscular destruction seen in dy/dy mice [123]. In 2012, Lefkowitz et al. reported that the administration of anti-RANKL reagent, denosumab, in FSHD patients improved muscle strength and dystrophic symptoms [124]. Recently, RANKL was reported to reduce muscular function when expressed in muscle cells. Anti-RANKL antibody treatment inhibits the NF-κB pathway and reduces muscle inflammation and damage in dystrophic mice [125]. Osteoprotegerin KO mice, which lack a secreted decoy receptor for RANKL, displayed reduced locomotor activity and signs of muscle weakness. Inhibiting RANKL improved the selective weakness and atrophy of fast-twitch IIb myofibers [126]. In addition, RANKL inhibition improved muscle strength and insulin sensitivity in osteoporotic mice and humans [127]. Therefore, caldecrin, by virtue of its anti-RANKL and anti-inflammatory activities, could be a suitable therapeutic approach for skeletal muscle dysfunction.

## 5. Concluding Remarks

We have highlighted the serum calcium-decreasing factor caldecrin, which was first discovered in the pancreas, and its structure and protease activity were identical to those of chymotrypsin C (CTRC). Protease-deficient caldecrin inhibits RANKL-stimulated osteoclast differentiation of BMMs and bone resorption mediated by mature osteoclasts. Additionally, caldecrin inhibits osteoclast differentiation stimulated by LPS and inflammatory M1 macrophage polarization stimulated by LPS and IFNγ through the TREM-2 pathway. Furthermore, caldecrin ameliorates the symptoms of several diseases, including osteoporosis, RA, and muscular dystrophy. Thus, caldecrin is a protease with chymotryptic hydrolysis activity and non-proteolytic functions, which modulate physiological and pathological bone metabolism and inflammation via the TREM2 pathway.

## Data Availability

Not applicable.
